# COLORECTAL CANCER: HISTOPATHOLOGICAL PROFILE AND PREVALENCE OF DNA REPAIR SYSTEM DEFICIENCY IN PATIENTS SUBMITTED TO SURGICAL TREATMENT IN A UNIVERSITY HOSPITAL

**DOI:** 10.1590/0102-672020230053e1771

**Published:** 2023-10-23

**Authors:** Julia Werner de Oliveira, Raquel Aguirra de Moraes, Samya Hamad Mehanna, Julia Costa Linhares

**Affiliations:** 1 Faculdade Evangélica Mackenzie do Paraná – Curitiba (PR), Brazil.; 2 Hospital Universitário Evangélico Mackenzie, Pathology Service – Curitiba (PR), Brazil.

**Keywords:** Microsatellite instability, Colorectal neoplasms, DNA repair enzymes, Instabilidade de microssatélites, Neoplasias colorretais, Enzimas reparadoras do DNA

## Abstract

**BACKGROUND:**

Part of colorectal cancer cases occurs due to modifications in the DNA mismatch repair system, which are responsible for microsatellite instability. This alteration results in an unconventional phenotypic pattern of colorectal cancer.

**AIMS:**

To describe the epidemiological, histopathological and molecular profiles of patients with colorectal cancer who underwent surgical treatment in a reference hospital.

**METHODS:**

This is a cross-sectional, retrospective study with a quantitative approach, that included a review of patients’ medical records who underwent oncological surgery for colorectal cancer.

**RESULTS:**

A total of 122 colorectal cancer cases were identified, with microsatellite instability detected in 8.2% of the sample. The gender distribution was similar, with 52.46% males, and the weighted average age was 63 years (standard deviation±11.65). However, in the microsatellite instability group, the predominant age was below 60 years. Regarding the histological type, adenocarcinoma not otherwise specified accounted for 80.33% of the cases, being the most prevalent in both groups, with the mucinous type being more frequent among the instability cases. The pT3 pathological staging (46.72%) was the most predominant. The topography was more prevalent on the left (60.66%), but there was a significant difference when compared to the group with microsatellite instability, in which 80% of the neoplasms were located on the right (p=0.006).

**CONCLUSIONS:**

Differences in age and neoplastic topography found in microsatellite instability samples highlight the distinctive presentation pattern of the disease. Recognizing these characteristics is essential for developing prevention strategies, in addition to early and accurate diagnosis of colorectal cancer.

## RESUMO

**RACIONAL:**

Parte dos casos de câncer colorretal ocorre devido a alterações nas enzimas de reparo do DNA, responsáveis pela instabilidade de microssatélites. Esta alteração resulta em um padrão fenotípico não convencional de câncer colorretal.

**OBJETIVOS:**

Descrever os aspectos epidemiológicos, histopatológicos e moleculares dos pacientes com câncer colorretal submetidos a tratamento cirúrgico em hospital de referência.

**MÉTODOS:**

Trata-se de um estudo transversal, retrospectivo com abordagem quantitativa, com revisão de prontuários médicos de pacientes submetidos a cirurgia oncológica por câncer colorretal.

**RESULTADOS:**

Foram registrados 122 casos de câncer colorretal, com instabilidade de microssatélites detectada em 8,2% da amostra. A distribuição por sexo foi semelhante, sendo 52,46% do sexo masculino, e média ponderada de idade de 63 anos (±11,65), contudo no grupo com instabilidade, a faixa etária predominante foi abaixo de 60 anos. Em relação ao tipo histológico, o adenocarcinoma sem outra especificação representou 80,33% dos casos, sendo o mais prevalente em ambos os grupos, mas com maior frequência do tipo mucinoso em caso de instabilidade. O estadiamento patológico pT3 (46,72%) foi predominante. A topografia da neoplasia foi mais prevalente à esquerda (60,66%), mas houve diferença significativa em relação ao grupo com instabilidade de microssatélites, no qual 80% das neoplasias localizavam-se à direita (p=0,006019).

**CONCLUSÕES:**

As diferenças de idade e topografia neoplásica encontradas nas amostras com instabilidade de microssatélites destacam o padrão não habitual de apresentação da doença. O conhecimento, portanto, dessas distinções é necessário para o desenvolvimento de estratégias de prevenção, além de diagnóstico precoce e preciso do câncer colorretal.

## INTRODUCTION

Colorectal cancer (CRC) is the second most common cancer in both sexes in Brazil, behind non-melanoma skin cancer. According to the National Cancer Institute (INCA), CRC had the third-highest mortality rate in 2019^24^. For each year of the 2020–2022 triennium, there were an estimated 20,540 CRC cases in males and 20,470 in females in the country^24^.

Most of these patients require surgical intervention for tumour resection; therefore, the anatomopathological characteristics of the neoplasm are critical for defining therapy. In addition, staging and patient prognosis are established from the tumour definition by the TNM system (T: tumor, N: lymph node, M: metastases)^27^.

Although cell replication is responsible for the maintenance of the organism, it depends on the deoxyribonucleic acid (DNA) replication — a process that can result in errors due to inadequate incorporation of bases. However, the organism has a process for repairing replication errors that, upon recognition, corrects them. This process is called DNA mismatch repair system or mismatch repair (MMR) and is encoded by the genes MLH1, MSH6, MLH2, and PMS2, among others, which together are called repair genes^7^.

The repair genes encode four homonymous proteins that function as heterodimers: MLH1-PMS2 and MSH2-MSH6. Thus, mutations or loss of function in these genes cause loss of protein expression and, consequently, in the deficiency of the repair process (dMMR), resulting in microsatellite instability (MSI)^5,7^.

According to recent research, approximately 15% of CRC patients have sporadic MSI^7,24^, with hypermethylation of the MLH1 promoter being the most common^7^. Thus, molecular testing for MSI is recommended for patients with CRC due to the impact on therapeutic planning^19^. Immunohistochemical analysis is the most accessible method to define the MSI status^4,21^.

The high rates of CRC incidence and mortality emphasize the need to provide accurate diagnoses for patients. Therefore, initiatives are needed to enhance knowledge and strategies for screening, prevention, and early detection, in addition to the identification of important pathological and genetic alterations, with emphasis on both loss of expression and deficiency in DNA repair enzymes^17^.

The purpose of this study was to describe the epidemiological, histopathological and molecular aspects of patients with CRC undergoing surgical treatment at a reference hospital in the state of Paraná.

## METHODS

This was a cross-sectional and retrospective study with a quantitative approach, conducted in the city of Curitiba, Paraná State. Medical records of patients were reviewed at the Hospital Universitário Evangélico Mackenzie (HUEM). Data collection occurred between November 2019 and June 2022 by gathering anatomopathological reports of surgical specimens and immunohistochemical studies to assess the state of DNA repair enzymes (MLH1, MSH2, MSH6, and PMS2) of CRC patients undergoing oncology surgery. Patients with missing data in their medical records and/or with incomplete immunohistochemical analyses were excluded. Several variables were considered, such as sex, age, location of the tumour, histological type, and the presence or absence of metastases.

For analysis, data were exported to a Microsoft Office Excel spreadsheet, where descriptive statistics using absolute numbers and percentages were used. The chi-square test was performed when applicable with a significance level of 0.05. The study was approved by the HUEM Research Ethics Committee under protocol n° 4,901,126, as required by Resolution n° 466/12 of the National Research Council, which regulates scientific research on humans. The data obtained were compared to the literature on this subject.

## RESULTS

From November 2019 to June 2022, a total of 122 CRC cases were registered at the hospital's high-complexity care centre and met the inclusion criteria for this study. Table 1 shows the sample characteristics distributed by sex. Males and females were distributed similarly, with a slight predominance of males, accounting for 52.46% of cases. Table 2 presents the distribution of CRC patients by age group (10-year interval). The age group between 61 and 70 years was the most predominant, accounting for 29.51% of all cases.

**Table 1:** Distribution of patients with colorectal cancer by sex (n=122).

Variables		n	%
Sex			
	Females	58	47.54
	Males	64	52.46

**Table 2:** Distribution of patients with colorectal cancer by age group (n=122).

Variables		n	%
Age group (years)			
	31–40	3	2.46
	41–50	20	16.39
	51–60	31	25.41
	61–70	36	29.51
	71–80	26	21.31
	81–90	6	4.92

Table 3 reveals the distribution of histopathological and topographic characteristics of CRC. Adenocarcinoma not otherwise specified (NOS) was found in 80.33% of cases. Regarding categorization according to location, tumours located on the right included the cecum, ascending colon, and transverse colon, while those on the left included the descending colon, sigmoid colon, and rectum, as described in the literature^9,16^. Tumours on the left were the most frequent, accounting for 60.66% of the sample, while those on the right accounted for 39.34%. Furthermore, 9.84% of patients received neoadjuvant treatment prior to the surgical procedure.

**Table 3:** Distribution of histopathological and topographical variables in patients with colorectal cancer (n=122).

Variables		n	%
Topography			
	Tumour on the right side	48	39.34
	Tumour on the left side	74	60.66
Histological type			
	Mucinous intestinal adenocarcinoma	21	17.21
	Intestinal adenocarcinoma (NOS)	98	80.33
	Others	3	2.46

NOS: not otherwise specified.

**Figure 1:**
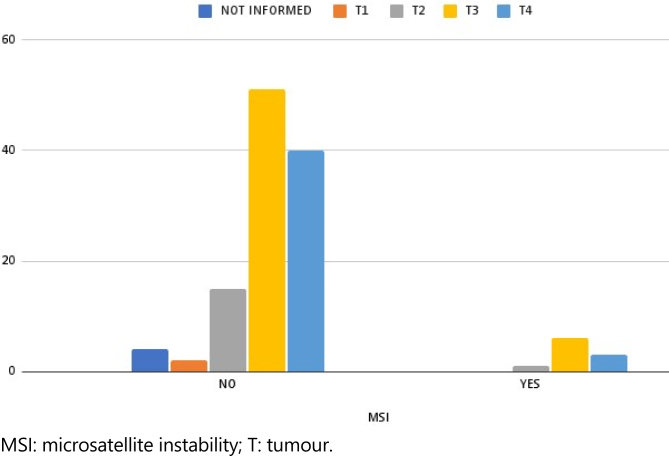
Microsatellite instability count according to tumoral extension.

Regarding TNM pathological staging, tumour characteristics, and depth of invasion of the neoplasm, pT3, which invades through the muscularis propria into pericolonic tissues, was predominant (46.72%), followed by pT4 (35.25%), which infiltrates the serous surface. Lesions classified as pT1 (submucosal invasion), and pT2 (muscularis propria invasion) represented a minority of cases (Figure 1).

**Figure 2:**
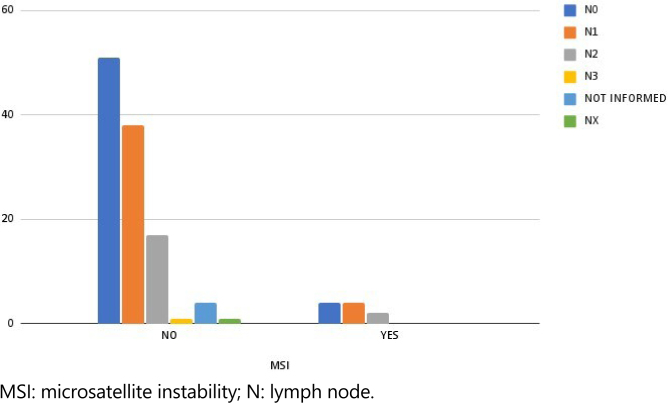
Microsatellite instability count according to lymph node metastases.

In regards to regional lymph node metastases (pN), pN1, which involves 1 to 3 lymph nodes, was detected in 34.43% of the sample, while pN2, which involves more than 4 lymph nodes, was found in 15.57%. Patients with no identified metastases (pN0) accounted for 45.08% of the sample, as shown in Figure 2.

Finally, 9.02% of the reports had data on the presence of distant metastases (pM1) with the liver being the most prevalent site.

Table 4 reveals the results of the immunohistochemical analysis of the search for DNA repair enzymes (MLH1, MSH2, MSH6, and PMS2). Cases in which there was preservation and positivity of antibodies were defined as microsatellite stability; cases of loss or abnormal immunoexpression were suggestive of MSI. Abnormal gene immunoexpression was found in 8.2% of the sample.

**Table 4:** Prevalence of DNA repair enzymes expression (MLH1, MSH2, MSH6, and PMS2) detected by immunohistochemistry in colorectal cancer patients (n=122).

Variables		n	%
MSI			
	No	112	91.80
	Yes	10	8.20

MSI: microsatellite instability.

Table 5 summarizes data on the sex and age of MSI patients. In the sample where MSI was detected, specifically, 70% were men, with no significant difference between sex (p=0.246). Furthermore, 60% of the tumours were NOS adenocarcinoma, which was most prevalent in the age group between 51 and 60 years, accounting for 40% of the sample.

**Table 5:** Distribution of sociodemographic variables in patients with colorectal cancer associated with microsatellite instability (n=10).

Variable			n					
**MSI count**			**Sex**					
	MSI		F			M		
		No	55			57		
		Yes	3			7		
**MSI count**			**Age**					
	MSI		31–40	41–50	51–60	61–70	71–80	81–90
		No	3	18	27	33	26	5
		Yes	0	2	4	3	0	1

MSI: microsatellite instability; F: female; M: male.

Table 6 shows the results of CRC topography and histological type in MSI patients. Regarding CRC topography, 80% of the tumours were located on the right (p=0.006), contrasting with the general sample. The most predominant histological type in the study sample was NOS adenocarcinoma, which appeared in six out of ten MSI cases, followed by mucinous, which appeared in three cases.

**Table 6:** Distribution of histopathological and topographic variables of patients with colorectal cancer associated with microsatellite instability (n=10).

Variables			n		
**MSI count**			**Location**		
	MSI		Tumour on the right	Tumour on the left	
		No	40	72	
		Yes	8	2	
**MSI count**			**Histological type**		
	MSI		Mucinous intestinal Adenocarcinoma	Intestinal-type Adenocarcinoma (NOS)	Others
		No	18	92	2
		Yes	3	6	1

MSI: microsatellite instability; NOS: not otherwise specified.

## DISCUSSION

The 122 CRC cases surgically treated in a high-complexity health centre were similar to other studies conducted in Brazil^3,9,10,23-26^. Results showed a slightly higher prevalence of males, accounting for 52.46% of cases. As for the 10 MSI patients, 3 were female and 7 were male.

According to the National Cancer Institute (INCA), in 2021, 9.2% of cancers, except non-melanoma skin cancers, were of colorectal origin in females and 9.1% in males. In terms of the absolute number of CRC cases, there was a slight predominance of males, with 20,540 new cases in 2020 compared to 20,470 females. According to the American Cancer Society (AMS)^1^, the estimated total number of new cases in 2022 also had a male predominance, with 80,690 new occurrences compared to 70,340 females.

Regarding the age group, a wide range was found, from 35 to 86 years, with a weighted average of 63 years (standard deviation±11.65). The age groups were also subdivided into 10-year intervals and evaluated (Table 2); 29.51% were between 61 and 70 years old.

Age is a risk factor for CRC development. According to the AMS, CRC is more prevalent after the age of 50, and the mean age of disease diagnosis is 72 years in women and 68 in men^18^. Furthermore, the risk of developing CRC increases significantly with each decade of life. According to INCA regarding the risk of CRC occurrence between 2016 and 2018, the probability was 0.4% for those aged between 0 and 49 years, and 3% for those aged 70 years and older^1^. In a Brazilian study^9^, of 521 cases of patients who underwent CRC surgery, the mean age was 63 years and there was a wider age range of occurrence (between 50 and 80 years), corroborating the higher disease prevalence in the older population observed in current studies.

Regarding tumour location, CRC can develop throughout the entire large intestine, including the cecum, ascending colon, transverse colon, descending colon, sigmoid colon, and rectum. Considering the general differences, these structures are typically segmented into the right and left colons, with the splenic flexure serving as a divider. The primary topography of CRC is a significant factor in treatment decision-making, in addition to being a prognostic indicator of the patient's survival^2,3,28^, since the sides are related to various distinct cancer genetic origins.

Loupakis et al.^16^ reported a higher prevalence of CRC on the left side, although the prognosis was worse for right-sided colon tumours. A study conducted at Hospital das Clínicas da Faculdade de Medicina da Universidade de São Paulo (HCFMUSP)^3^, reported that cases of colon cancer on the right side had a significant difference in the age of the individual at diagnosis. The disease were at higher advanced stage at the time of surgery and had a higher occurrence of the mucinous histological type. Another important distinction concerns symptomatology — although cancers on the right side have milder symptoms, such as unnoticed bleeding, which can delay diagnosis, cancers on the left side cause more frequent changes in bowel habits, intestinal transit changes, and obstructions^3,13,31^. In the analysis of the 122 cases in this study, there was a prevalence of neoplasm on the left side in 60.66% of the individuals; 39.34% on the right side.

Regarding tumour histological type, the NOS was identified in 98 analyses, accounting for 80.33% of the cancers, followed by the mucinous type, which accounted for 17.21%. TNM staging is based on the level of intestinal wall penetration, the number of affected lymph nodes, and the presence or absence of distant metastases^26^. It is the most important pathological classification in all international CRC guidelines and, therefore, the gold standard classification for disease prognosis^6^, in addition to being relevant for patient-specific therapy planning^23,24^.

The prevalence of more advanced TNM staging found in this study is in agreement with the literature and with similar studies conducted in other centres^9,10,25^. This finding is concerning because late diagnosis reduces the cure rate for the disease^10^. In this regard, since CRC only becomes symptomatic in advanced stages, structured screening programs using colonoscopy have been implemented worldwide, with the objective of increasing early detection of CRC and reducing its morbimortality^8^.

Colorectal carcinoma has its main genesis in the evolution of a dysplastic polypoid structure. Polyps are tumour masses that protrude into the intestinal lumen as a result of mucosal proliferation due to inflammatory stimuli or tissue architecture disorder. Polyps can be classified as non-neoplastic, such as hyperplastic, hamartomatous, inflammatory, and lymphoid. On the contrary, adenoma-type polyps, which develop from epithelial proliferative dysplasia, are considered precursors of carcinoma. The prevalence of adenomas increases with age, affects both genders similarly, and is associated with a hereditary predisposition to sporadic forms. Histologically, adenomas can be classified into three subtypes: tubular, villous, and tubulovillous^20^. The risk of malignant transformation is independently associated with polyp size, histological architecture, and degree of dysplasia. High-grade dysplasia is frequently found in villous areas^14^.

The development of CRC can occur through two distinct genetic mechanisms: one involving chromosomal instability, which occurs in approximately 85% of typical cases, and the other involving MSI, which occurs in 15 to 20% of cases^11,17,21^. Microsatellites are normal segments of DNA with repetitive sequences of nucleotides of defined length. Therefore, the insertion or deletion of nucleotides that causes changes in the number of repetitions or the length of the segment is known as MSI^5^. This phenomenon is related to the MMR genes, which encode four homonymous proteins that function as heterodimers: MLH1-PMS2 and MSH2-MSH6. Thus, mutations or loss of function in these genes result in the loss of expression of repair proteins and, consequently, in a dMMR, which results in MSI^7^.

The comprehension of this process explains why MSI can be assessed not only through polymerase chain reaction (PCR) but also through immunohistochemical analysis, with evidence that the latter configures a straightforward and more accessible method and, thus, a viable alternative to PCR. Immunohistochemical analysis is performed using antibodies, mainly anti-MLH1 and anti-MLH2, as well as anti-MSH6 and anti-PMS2, to diagnose the non-expression of MMR proteins (dMMR) and therefore the MSI^12,15,21,29^.

The mechanism of tumour genesis was the objective of this research. However, a prevalence of only 8.2% was found which is not in agreement with previous studies. This discrepancy may be explained by the limited sample size, as there is a higher prevalence of tumours on the left side, where instability is less frequent.

In cases of cancer associated with MSI, the mucinous histological type is the most predominant^17^. In this study sample, the most predominant histological type was NOS adenocarcinoma, followed by mucinous. However, when the percentages in the overall sample were considered, mucinous adenocarcinoma was more frequent in cancers associated with MSI.

Tumours arising from the pairing error pathway occur mostly in the proximal colon and normally affect younger patients^8,22,30^. Although the left colon was the most common site in this sample, when we considered only the cases with MSI, 80% of the tumours were located in the right colon and 20% in the left, with a significant difference in topography between the groups with and without MSI. As for age, the majority of MSI patients were between 51 and 60 years old, followed by those between 41 and 50 years old. These findings are in agreement with current research, which shows a higher incidence of this genetic error in patients under the age of sixty^4,8,13,22,30,31^.

CRC is a public health concern due to its high national and global prevalence, with the majority of cases having a sporadic origin. This study enabled the description of the epidemiological profile of CRC patients with findings comparable to previous studies, including parity between female and male genders and primary location in the rectosigmoid junction.

## CONCLUSIONS

The histopathological evaluation indicated a predominance of tubular adenocarcinoma in the pT3 and pN0 stagings. In addition, 8.2% of the patients had a deficiency in the DNA repair system detected by immunohistochemistry, indicating MSI, and there was a difference in the topographic profile of the CRC in the presence of MSI, with MSI being more frequent on the right side of the intestine.

This study's panorama incorporates elements from the medical sciences, providing important characteristics about this population group. The goal is the development of preventive strategies, as well as early and accurate CRC diagnosis.
